# Phylogenetic analyses of the polyprotein coding sequences of serotype O foot-and-mouth disease viruses in East Africa: evidence for interserotypic recombination

**DOI:** 10.1186/1743-422X-7-199

**Published:** 2010-08-23

**Authors:** Sheila N Balinda, Hans R Siegismund, Vincent B Muwanika, Abraham K Sangula, Charles Masembe, Chrisostom Ayebazibwe, Preben Normann, Graham J Belsham

**Affiliations:** 1 Makerere University, Institute of Environment and Natural Resources P.O. Box 7298, Kampala, Uganda; 2 Department of Biology Ole Maaløes Vej 5, DK-2200, Copenhagen N, Denmark; 3 National Veterinary Institute, Technical University of Denmark, Lindholm, DK-4771 Kalvehave, Denmark; 4 Ministry of Agriculture Animal Industry and Fisheries, P.O. Box 513, Entebbe, Uganda; 5 Foot-and-Mouth Disease Laboratory, Embakasi, P. O. Box 18021, 00500, Nairobi, Kenya

## Abstract

**Background:**

Foot-and-mouth disease (FMD) is endemic in East Africa with the majority of the reported outbreaks attributed to serotype O virus. In this study, phylogenetic analyses of the polyprotein coding region of serotype O FMD viruses from Kenya and Uganda has been undertaken to infer evolutionary relationships and processes responsible for the generation and maintenance of diversity within this serotype. FMD virus RNA was obtained from six samples following virus isolation in cell culture and in one case by direct extraction from an oropharyngeal sample. Following RT-PCR, the single long open reading frame, encoding the polyprotein, was sequenced.

**Results:**

Phylogenetic comparisons of the VP1 coding region showed that the recent East African viruses belong to one lineage within the EA-2 topotype while an older Kenyan strain, K/52/1992 is a representative of the topotype EA-1. Evolutionary relationships between the coding regions for the leader protease (L), the capsid region and almost the entire coding region are monophyletic except for the K/52/1992 which is distinct. Furthermore, phylogenetic relationships for the P2 and P3 regions suggest that the K/52/1992 is a probable recombinant between serotypes A and O. A bootscan analysis of K/52/1992 with East African FMD serotype A viruses (A21/KEN/1964 and A23/KEN/1965) and serotype O viral isolate (K/117/1999) revealed that the P2 region is probably derived from a serotype A strain while the P3 region appears to be a mosaic derived from both serotypes A and O.

**Conclusions:**

Sequences of the VP1 coding region from recent serotype O FMDVs from Kenya and Uganda are all representatives of a specific East African lineage (topotype EA-2), a probable indication that hardly any FMD introductions of this serotype have occurred from outside the region in the recent past. Furthermore, evidence for interserotypic recombination, within the non-structural protein coding regions, between FMDVs of serotypes A and O has been obtained. In addition to characterization using the VP1 coding region, analyses involving the non-structural protein coding regions should be performed in order to identify evolutionary processes shaping FMD viral populations.

## Background

**Figure 1:**
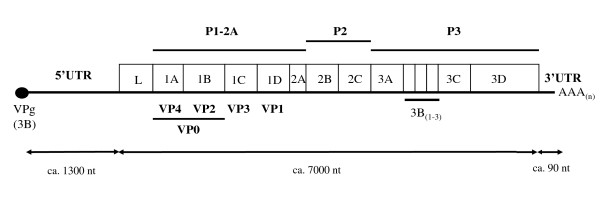
**Genomic structure of FMDV**. Genome organization of FMD viruses modified from [1]. The 5'untranslated region (ca. 1300 nt.), polyprotein region (ca. 7000 nt.) and the 3'untranslated region (ca 90 nt.) are shown. P1-2A, P2 and P3 indicate the precursors or intermediates for the capsid region proteins, 2BC proteins and the 3ABCD proteins, respectively.

Foot-and-mouth disease virus (FMDV) is a member of the *Picornaviridae *family, belonging to the genus *Aphthovirus *[1] and is the causative agent of foot-and-mouth disease (FMD), a highly contagious infection of cloven-hoofed domestic animals and over 70 wildlife species [2]. The viral genome is a positive-sense single stranded RNA (about 8.3 kb, see Figure 1), encoding a polyprotein which is processed to yield structural and non-structural proteins [1]. The RNA genome undergoes a high rate of mutation due to error prone replication by the RNA polymerase resulting in high genetic diversity [3], however, not all coding regions evolve at the same rate [4]. The structural protein coding region, particularly the sequence for VP1, has been shown to vary significantly between strains and serotypes hence it is used extensively for evolutionary relationship inference [5]. Persistent infection, recombination, and quasi-species dynamics have also been suggested as contributing to the genetic variation [6-8]. Globally, the virus exists in seven distinct serotypes; the Southern African territories [SAT] types 1-3 and Eurasian types namely O, A, C and Asia 1. Immunity to one serotype does not confer protection against another. In Africa, FMD is endemic in the sub-Saharan region with six of the known serotypes recorded in the Eastern African region [9]. Of the numerous outbreaks reported in this region, most are attributed to serotype O, followed by A, SAT 2 and SAT 1 but some cases of serotype C have been reported in Ethiopia, Kenya and Uganda [9]. SAT 3 has been isolated in 1970 and 1997 from African buffalo in the Queen Elizabeth National Park (Uganda) but has otherwise not been recorded elsewhere in East Africa [9,10]. Previous studies, based on VP1 coding sequences, have shown that four different lineages (EA 1-4) of type O FMDV are present in this region[11]. This complex intra-serotypic variation coupled with the presence of multiple serotypes has complicated disease control, which is achieved mainly by vaccination and restrictions on animal/animal product movement. In East Africa, most molecular studies have sought to identify phylogenetic relationships among viruses relying on the sequence of the VP1 coding region [12]. Although used extensively for phylogenetic studies, the VP1 sequence alone may be inadequate for revealing all processes shaping diversity and evolution of the viruses in the region [13-15].

Elsewhere on the African continent, remarkable progress in FMD molecular epidemiology has been made particularly in Southern Africa, a region also characterized by SAT type endemicity. Molecular epidemiological studies have been able to reveal the origin and routes of FMD transmission in this region. In addition, the important epidemiological role of the African buffalo (*Syncerus caffer*) in maintenance of this virus and transmission to other cloven-hoofed animals has been highlighted [16-18].

In this study, we have analyzed the polyprotein coding sequence (6719 nt) of serotype O FMD viruses to get insights into relationships among these viruses to infer processes shaping their diversity in the East African region. Sequences analyzed in this study include isolates from Kenya and Uganda obtained between the years 1992 and 2006 and sequences already in the Genbank database.

## Materials and methods

### Viral isolates

The viruses investigated in this study were collected during outbreaks in the indicated years in Kenya (K) and Uganda (U). Four samples; O/K/52/1992, O/K/117/1999, O/K/109/2000 and O/K/48/2005 were from Kiambu, Nakuru, Uasin Gishu and Kiambu districts, respectively. FMDV RNA was extracted from these samples following virus propagation in baby hamster kidney (BHK) cells. The O/U/25/2006 (Mpigi district) sequence was generated from RNA prepared directly from an oropharyngeal sample while the O/U/312/2006 virus was isolated from an orophyrangeal sample from Mbarara district and grown in primary bovine thyroid (BTY) cells.

### RNA extraction, cDNA synthesis, PCR and Cycle sequencing

RNA was extracted from virus harvests and directly from orophyrangeal fluid using the QIAamp^® ^Viral RNA kit (Qiagen, Hilden, Germany) according to the manufacturer's instructions. The cDNA synthesis was carried out using Ready-To-Go™ You-Prime First - Strand Beads (GE Healthcare Life Sciences, Sweden) with random hexamer primers (pdN_6_).

PCR reactions of overlapping fragments were performed in a final volume of 50 μl using 2-5 ng of cDNA, 0.2 pmol of primers (Table 1) and 2.5 U of AmpliTaq gold DNA polymerase (Applied Biosystems), 200 μM of each dNTP (dATP, dCTP, dGTP, dTTP) and 1.5 mM MgCl_2_. Following the activation of AmpliTaq gold DNA polymerase at 95°C for 5 min, reaction mixtures were denatured at 95°C for 15 s followed by 60°C for 2 min to allow for primer annealing. For each cycle, a chain elongation step at 72°C for 1 min 20 s was allowed. This process was repeated 30 times and final extension continued at 72°C for 5 min. The resultant PCR products were analysed using 2% agarose gel electrophoresis with a molecular weight marker (ΦX174-RF DNA, Amersham Biosciences). Purification of the PCR products to remove oligonucleotides, dNTPs and enzyme was achieved with a QIAquick^® ^PCR purification kit (Qiagen, Hilden, Germany). Sequencing was performed in both directions using a Big dye Terminator V 3.1 kit (Applied Biosystems) and ran on an automated DNA Sequencer (ABI PRISM^® ^3700) by Macrogen, Korea, using the same primers as employed in the PCRs

**Table 1:** Summary of primers used

Primer ID	Primers	Fragment/Primer combination
8-A PN 2	GTCNCCTATTCAGGCNTAGAAG	Fragment 1: 8-A PN-35 & 8-A PN-2
8-A PN 3	GGCTAAGGATGCCCTTCA	
8-A PN 4	AACCAGTCNTTCTTNTGNGTG	Fragment 2: 8-A PN 3 & 8-A PN-4
8-A PN 6	CCNGTNNACATGAANTGCAGGTT	
8-A PN 14	ATCTCNAACTCAAACACTCTG	Fragment 3: 8-A PN 34 & 8-A PN-83
8-A PN 22	AAGGACCCNGTCCTTGTGGC	
8-A PN 23	CCGTCNAAGTGGTCAGGGTC	Fragment 4: 8-A PN 51 & 8-A PN-6
8-A PN 34	ATGGACACNCAGCTTGGTGAC	
8-A PN 35	GAGAAANGGGACGTCNGCGC	Fragment 5: 8-A PN 84 & 8-A PN-85
8-A PN 45	GGAAGAAACTCGAGGCGAC	
8-A PN 46	TGGTCGTTTGCCTCCGTGG	Fragment 6: 8-A PN 98 & 8-A PN-64
8-A PN 51	CCACAGATCAAGGTGTATGC	
8-A PN 64	GGTTGGACTCCACATCTCC	Fragment 7: 8-A PN 86 & 8-A PN-45
8-A PN 68	GGGTCCTTCAGCTGGTGG	
8-A PN 80	GGAGTGTTTGGCACTGCC	Fragment 8: 8-A PN 22 & 8-A PN-23
8-A PN 83	CTTTGGAAGGGAACTCACC	
8-A PN 84	TACTACACCCAGTACAGCG	Fragment 9: 8-A PN 46 & 8-A PN-87
8-A PN 85	TGCAGCTTCGGGTGCTCC	
8-A PN 86	GGCCATCCACCCGAGTG	Fragment 10: 8-A PN 99 & 8-A PN-68
8-A PN 87	CTCAAAGAATTCAATTGCTGC	
8-A PN 98	GCATCCACTTACTACTTTGC	Fragment 11: 8-A PN 113 & 8-A PN-14
8-A PN 99	TGTACCANCTTGTTNANGAGGTG	
8-A PN 101	CAGGGTTGAACACACCGAG	Fragment 12: 8-A PN 80 & 8-A PN-101
8-A PN 113	CGCGANACTCGCAAGAGAC	

### Sequence Analysis

Sequencher software 4.8 (Gene Code Corporation) was used to assemble the 12 overlapping fragment sequences generated per sample. Multiple alignments by log-expectation comparison were carried out using MUSCLE [19] incorporated within Geneious 4.7.6 software [20]. Phylogenetic analyses involving the determination of models of evolution were performed using hierarchical likelihood-ratio test of 24 models using PAUP*(v. 4.0 beta 10) [21] and MrModeltest (v 2.2) [22]. The GTR+I+G model was used and Bayesian inference analysis performed using MrBayes (v.3.1.2) [23] with the following settings: maximum likelihood model was six substitution types (nst = 6), with base frequencies set to variable values ("statefreqpr = dirichlet(1,1,1,1"). Rate variation across sites with a proportion of invariable sites was modeled using a gamma distribution (rates = invgamma). The Markov Chain Monte Carlo search was run with 4 chains for 500,000 generations with trees sampled every 100 generations; the first 1250 were discarded as burnin [23]. Recombination between sequences was analysed using SimPlot method 2.5 software [24].

## Results

### Sequence Characteristics and Phylogenetic Relationships

Almost the entire polyprotein coding sequence (6719 nt) of five FMDV isolates and a single Ugandan FMDV RNA sample were determined. Four of these samples had origins from the districts of Kiambu, Nakuru and Uasin Gishu in Kenya (from 1992, 1999, 2000 and 2005 respectively) while the two Ugandan isolates were from Mpigi and Mbarara districts (in 2006). All isolates belong to serotype O with percentage nucleotide sequence identities of the polyprotein coding region with representatives of six FMDV serotype as follows; O(89%), A(83%), C(84%), SAT1(73%), SAT2(77%) and SAT3(73%). The generated sequences have further been compared with sequences published in Genbank including the sequence of another recent Ugandan type O isolate from Kasese district (Accession no. EF611987[25]), which was determined previously. Twenty one additional serotype O sequences previously isolated from Europe, Asia, South America and Africa were included in the analysis. Furthermore, sequences from serotypes A, C, SAT 1, SAT 2 and SAT 3 were included for comparative purposes.

**Figure 2:**
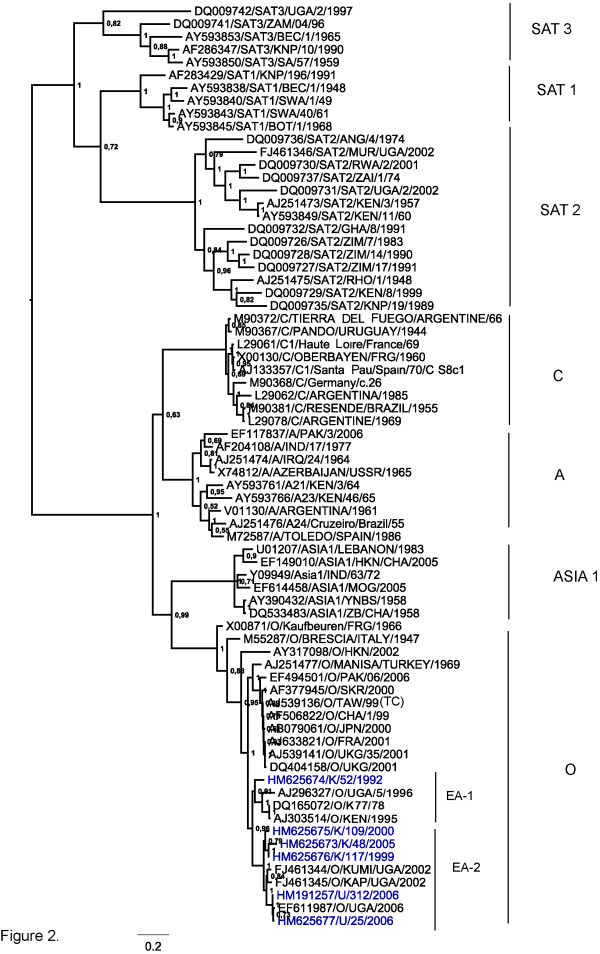
**Phylogenetic relationships of the VP1 coding region of type O East African viruses in comparison to other FMD virus serotypes**. Evolutionary relationships of the VP1 coding region of FMD type O viruses from East Africa are shown in comparison to other FMD virus serotypes. The tree was estimated using Bayesian inference analysis (MrBAYES) and percentage node values represent posterior probabilties (values that concentrate on a single tree when data is informative given a specified model of evolution for a particular sequence data set).

Phylogenetic relationships have been determined using the VP1 coding region for serotype identification. Furthermore, analyses using the coding regions for the Leader (L) protease, the whole capsid precursor (P1-2A) and for the non-structural protein precursors P2 and P3 individually, as well as for almost the entire polyprotein, have been performed. The phylogenetic relationships identified between these East African serotype O strains and representatives of each of the different FMDV serotypes for the VP1 coding regions are shown in Figure 2. The recent strains from Kenya and Uganda analysed here, each clearly belong to serotype O within topotype EA-2 while the older strain K/52/1992 belongs to topotype EA-1. These recent viral isolates each belong to a single evolutionary lineage albeit within different sub-lineages.

**Figure 3:**
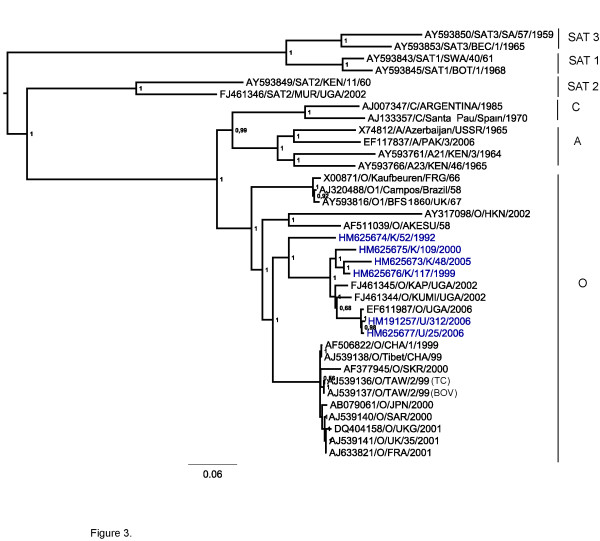
**Phylogenetic relationships derived for the polyprotein coding region**. Evolutionary relationships of the type O East African strains based on the polyprotein coding region (6719 nt) in comparison with selected sequences of type O from Asia, Europe and South America. Sequences of serotypes A, C, SAT 1, 2 and 3 are also included. Trees were estimated as for Figure 2.

Figure 3 shows the inferred phylogenetic relationships between serotype O viruses for the polyprotein coding region. In this phylogeny, with the exception of K/52/1992 which belongs to EA-1, the other East African strains are grouped into a single lineage which is distinct from the serotype O viruses isolated from Asia, Europe and South America. Similar phylogenies were observed for the Leader protease and the entire capsid precursor (P1-2A) (data not shown).

**Figure 4:**
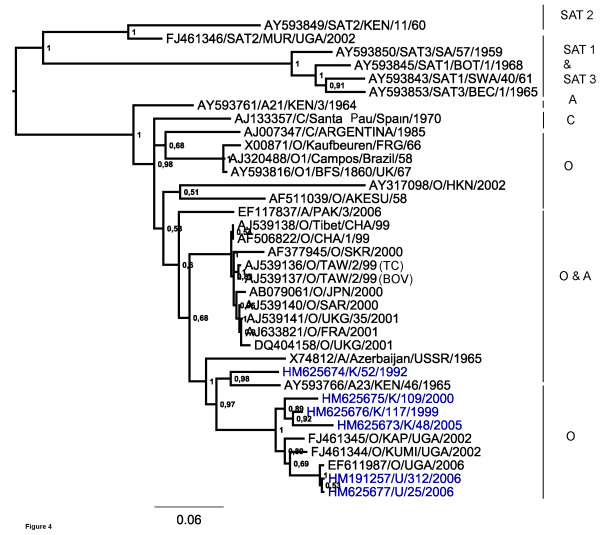
**Phylogenetic relationships derived for P2 coding regions**. Evolutionary relationships of the type O East African strains based on the P2 coding region with selected sequences of type O from Asia, Europe and South America. Trees were estimated as in Figure 2.

**Figure 5:**
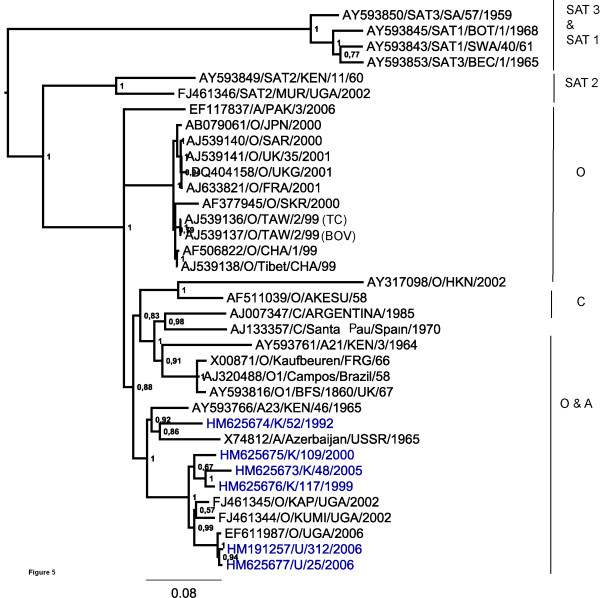
**Phylogenetic relationships derived for P3 coding regions**. Evolutionary relationships of the type O East African strains based on the P3 coding region with selected sequences of type O from Asia, Europe and South America. Trees were estimated as in Figure 2.

Figures 4 and 5 show the phylogeny inferred from the coding regions for the P2 (2BC) and P3 (3ABCD) precursors respectively. The P2 coding region follows a similar pattern as for the other coding regions of the East African strains with the exception that the isolate K/52/1992 showed a close relationship in this region to the sequence of a serotype A isolate (Accession no. AY593766, A23/KEN/46/65) obtained in 1964 from Kenya. Furthermore, within the P3 coding region, the 1992 Kenyan isolate is most closely related to a serotype A isolate obtained in 1965 from Azerbaijan in the former USSR (Accession no. X74812) and to the A23/KEN/1965 strain. It should be noted that in the other regions of the genome these serotype A viruses were most closely related to other serotype A virus strains (see Figures 2 and 3). These observations suggested that recombination may have occurred at some time between serotype O and A viral strains.

### Detection of Recombination

**Figure 6:**
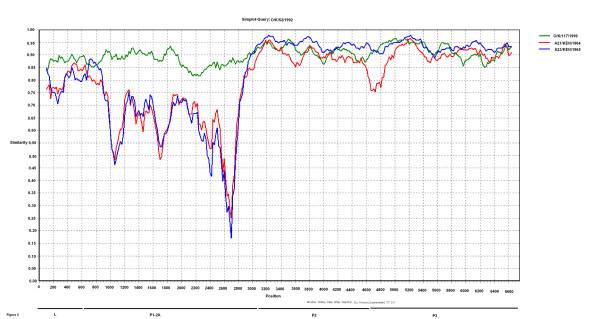
**Detection of recombination between serotypes O and A using Simplot analyses**. Recombination analyses using SimPlot 2.5 software (Ray, 1999). K/52/1992 was queried against AY593766/A/KEN/1965 (blue), AY593761/A/KEN/1964 (red) and K/117/1999 (green). Similarity plot with the Y-axis showing proportion of nucleotide identity and the X-axis showing the nucleotide positions along the alignment.

**Figure 7:**
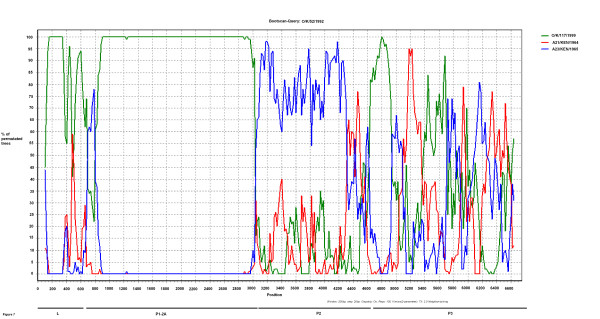
**Detection of recombination between serotypes O and A using bootscan analyses**. Analyses were performed as in Figure 6. Bootscan plot with the Y-axis showing percentage permutated trees while the X-axis shows nucleotide positions along the alignment

As indicated above in Figures 4 and 5, comparison of the genome sequences within the P2 and P3 coding regions showed that the O/K/52/1992 virus sequence in these regions was most closely related to some serotype A viruses although elsewhere within the genome this Kenyan virus was most closely related to other O serotype viruses. In order to establish possible recombination events further analyses were performed using similarity and bootscan plots (see Figure 6 and 7). The similarity and bootscan plots show the pairwise genetic similarities and bootscan values between a query sequence (O/K/52/1992) and selected strains of serotypes A and O (AY593766/A23/KEN/1965, AY593761/A21/KEN/1964 and O/K/117/1999) using a window size of 200 in steps of 20 nucleotides along the alignment. The examination of points at which similarities between the query, O/K/52/1992 and the test sequences increased or decreased, identified approximate break points. Figure 6 shows that the O/K/52/1992 sequence is most closely related to serotype O within the capsid region but has similar levels of percentage identity to both serotypes O and A within P2 and P3 coding regions. The Bootscan analysis (Figure 7) shows this query sequence is closely related to A23/KEN/1965 within the P2 region and has a mosaic pattern of similarity to both O/K/117/1999 and A21/KEN/1964 within the P3 coding regions.

## Discussion

Sequencing of these recent viruses from Kenya and Uganda has allowed for the assessment of variation of the East African isolates across almost the entire coding region relative to viruses from Asia, Europe and to a smaller extent South America. These East African viruses belong to serotype O based on the sequence of the VP1 coding region (Figure 2) and are part of a single evolutionary lineage (topotype EA-2) except for K/52/1992 (topotype EA-1). The related virus strains U/312/2006 and O/UGA/2006 (accession no. EF611987) [25] have also been identified as serotype O by antigen ELISA (data not shown).

Within the East African lineage, two major divisions are observed corresponding to their geographical locations with the Ugandan isolates comprising one sub-lineage and the Kenyan strains another. In addition, the Ugandan viruses are grouped in a temporal manner with the Uganda (2006) isolates clustering together but separate from the Uganda (2002) isolates. The East African lineage appears to have evolved independently from other serotype O viruses which are circulating globally. From comparisons of published serotype O sequences, three other main lineages were observed (Figure 3). An Asian lineage includes O/Akesu/1958 and O/HKN/2002 while a second lineage, widely referred to as the PanAsia I lineage [26], includes recent European O and Asian strains, the PanAsia II lineage (Figure 2) is represented by a viral strain from Pakistan (EF494501/O/PAK/06/2006) collected in the year 2006. The last lineage is comprised of earlier European strains from 1966 and 1967 together with the Campos strain from South America, indicative of intercontinental transmission between the Asian continent and Europe as well as South America and Europe.

Phylogenetic relationships of the P2 and P3 coding regions placed the K/52/1992 strain in a close relationship with serotype A viruses (from Kenya in 1965 and Azerbaijan/USSR/1965) (Figures 4 and 5). This points to the possibility that this strain may have arisen by recombination between viruses of different serotypes, a process seen within many picornaviruses both in nature, e.g. for poliovirus, Human enterovirus B [6,27-29], and in the laboratory including with FMD viruses [28]. The mosaic pattern observed within the P3 coding region may suggest recombination although this could also indicate genetic convergence. Earlier studies have shown that inter serotypic genetic exchange occurs more frequently within the Euro-asiatic viruses in comparison to amongst SATs or even between SAT and non-SAT viruses [8].

It is noteworthy that the most prevalent serotypes in the East Africa region are O followed by A [9] strongly suggesting the possibility of co-infection with both or even more serotypes within some animals.

The findings reveal the complexity of FMDV evolution, consistent with earlier studies that have shown that recombination is mainly restricted to non-structural coding regions.

This study therefore supports recombination as an evolutionary force causing genetic diversity within FMDV and shows the need for full genome analyses to identify such events.

## Competing interests

The authors declare that they have no competing interests.

## Authors' contributions

SNB	Study design, field work, laboratory work and data analyses, manuscript write-up. HRS Project design, manuscript preparation and proof reading. VBM Study design, manuscript preparation and proof reading. AKS Provided Kenyan viral isolates, laboratory work and manuscript proof reading. CM Field work, laboratory studies, manuscript proof reading. CA Field work, manuscript proof reading. PN Laboratory studies and generated sequence U/312/2006. GJB Study design, laboratory studies, manuscript preparation, proof reading and review. All authors have read and approved the final manuscript.
